# Chemoreception Regulates Chemical Access to Mouse Vomeronasal Organ: Role of Solitary Chemosensory Cells

**DOI:** 10.1371/journal.pone.0011924

**Published:** 2010-07-30

**Authors:** Tatsuya Ogura, Kurt Krosnowski, Lana Zhang, Mikhael Bekkerman, Weihong Lin

**Affiliations:** Department of Biological Sciences, University of Maryland, Baltimore County, Baltimore, Maryland, United States of America; University of Oldenburg, Germany

## Abstract

Controlling stimulus access to sensory organs allows animals to optimize sensory reception and prevent damage. The vomeronasal organ (VNO) detects pheromones and other semiochemicals to regulate innate social and sexual behaviors. This semiochemical detection generally requires the VNO to draw in chemical fluids, such as bodily secretions, which are complex in composition and can be contaminated. Little is known about whether and how chemical constituents are monitored to regulate the fluid access to the VNO. Using transgenic mice and immunolabeling, we found that solitary chemosensory cells (SCCs) reside densely at the entrance duct of the VNO. In this region, most of the intraepithelial trigeminal fibers innervate the SCCs, indicating that SCCs relay sensory information onto the trigeminal fibers. These SCCs express transient receptor potential channel M5 (TRPM5) and the phospholipase C (PLC) β2 signaling pathway. Additionally, the SCCs express choline acetyltransferase (ChAT) and vesicular acetylcholine transporter (VAChT) for synthesizing and packaging acetylcholine, a potential transmitter. In intracellular Ca^2+^ imaging, the SCCs responded to various chemical stimuli including high concentrations of odorants and bitter compounds. The responses were suppressed significantly by a PLC inhibitor, suggesting involvement of the PLC pathway. Further, we developed a quantitative dye assay to show that the amount of stimulus fluid that entered the VNOs of behaving mice is inversely correlated to the concentration of odorous and bitter substances in the fluid. Genetic knockout and pharmacological inhibition of TRPM5 resulted in larger amounts of bitter compounds entering the VNOs. Our data uncovered that chemoreception of fluid constituents regulates chemical access to the VNO and plays an important role in limiting the access of non-specific irritating and harmful substances. Our results also provide new insight into the emerging role of SCCs in chemoreception and regulation of physiological actions.

## Introduction

Sensory detection of the surrounding environment requires vertebrate specialized sensory organs to have access to external stimuli. In visual and auditory systems, distinct mechanisms control the access of specific stimuli to the eye and inner ear, and limit non-specific stimulation [1]. For example, the pupillary light reflex in mammalian eyes controls the amount of light reaching the retina, allowing photoreceptors to detect a wide range of light intensity as well as protecting the retina from burning sunlight [2]. While such regulation is well documented in visual and auditory systems [2], [3], little is known about whether and how the access of chemical stimuli to the VNO in the olfactory system is regulated. The VNO houses chemosensory neurons that detect pheromones and other semiochemicals [4], [5], [6], [7], [8], [9] and provide sensory information to regulate innate social and reproductive behaviors [7], [9], [10], [11], [12], [13], [14]. VNOs of reptiles are also involved in detecting chemicals from predators and prey [15]. In vertebrates, the structure of the VNO is well conserved. Each VNO contains a sensory epithelium and cavernous vessels. These tissues are enclosed in a bony tube, which has only one anterior opening to allow stimuli to enter the organ [15], [16], [17]. Semiochemical detection by physically isolated sensory neurons in the VNO generally requires animals to make contact with and draw in external stimulus fluids, such as urine and other bodily secretions, which are rich in semiochemical cues [18], [19], [20], [21]. The intake of stimulus fluids relies on the vasomotor movement of the cavernous vessels acting as a pumping mechanism [22], [23], [24]. Because the movement is controlled by the autonomic nervous system, which is activated when animals encounter novel conditions regardless of fluid contents [25], various chemicals including fluorescent dye and isotopically labeled amino acids, readily gain access to VNOs [21], [26], [27]. It is plausible that, in the absence of regulation, irritating and harmful substances present in aged and contaminated stimulus sources can gain greater access to the VNO and cause damage to the vomeronasal neurons. Also, because volatile pheromones blown to the nasal cavity can activate the VNO-accessory olfactory system [22], high levels of inhaled odorants diffusing into nasal mucus can also be pumped into the organ. Odorants at high levels are irritating to humans and animals [28], [29]. Thus it is essential to monitor fluid constituents to limit the VNO access of irritating and harmful substances.

Trigeminal peptidergic nerve fibers, containing substance P, provide sensory input about noxious chemicals in the nasal cavity. These fibers are generally believed to be free nerve endings reacting directly with chemical stimuli [30]. However, the discovery of SCCs in the mammalian respiratory tract has challenged this belief. The SCCs express signaling components known to mediate taste signal transduction, such as bitter taste receptors, α-gustducin and TRPM5. Many SCCs are trigeminally innervated and respond to chemical stimuli [31], [32], [33], [34], [35], [36]. Previously, when we studied the TRPM5 expression in the olfactory sensory neurons [37], [38] and in SCCs of the respiratory epithelium [35], we noticed numerous TRPM5-expressing SCCs in the VNO entrance duct, the only pathway for stimulus fluids to enter the VNO.

We sought to investigate the role of the SCCs in detecting chemical stimuli and regulating access to the VNO. We determined the SCC's distribution in the VNO, their expression of chemosensory signaling proteins, and their physiological responses to chemical stimuli using immunolabeling and physiological recordings. We found that the SCCs reside in an appropriate location for detecting the chemical constituents in fluids destined to the VNO lumen. The SCCs also express a number of key signaling proteins and responded to various odorous irritants and bitter-tasting substances. In addition, we developed a quantitative dye assay to estimate the access of stimulus fluids to the VNOs of behaving mice. We found that the amounts of dye-stimulus mixtures in the VNOs are dependent on the stimuli and their concentrations. Further, we determined sensory signaling transduction mechanisms using both TRPM5 knockout mice and inhibitors to block the activity of TRPM5 and PLC. We found that the PLC expressed in the SCCs plays an important role in detecting both odorous and bitter substances, and that a functioning TRPM5 is critical for signal transduction of bitter substances, consequently, limiting their access to the VNOs. Taken together, our results demonstrate that chemoreception of fluid constituents regulates the chemical access to the VNO and that SCCs of the VNO, especially those residing at the entrance duct, play an important role in this chemoreception-mediated regulation. Preliminary results of this study have been published in abstract form [39], [40].

## Results

### SCCs of the VNO reside preferentially at the entrance duct

**Figure 1:**
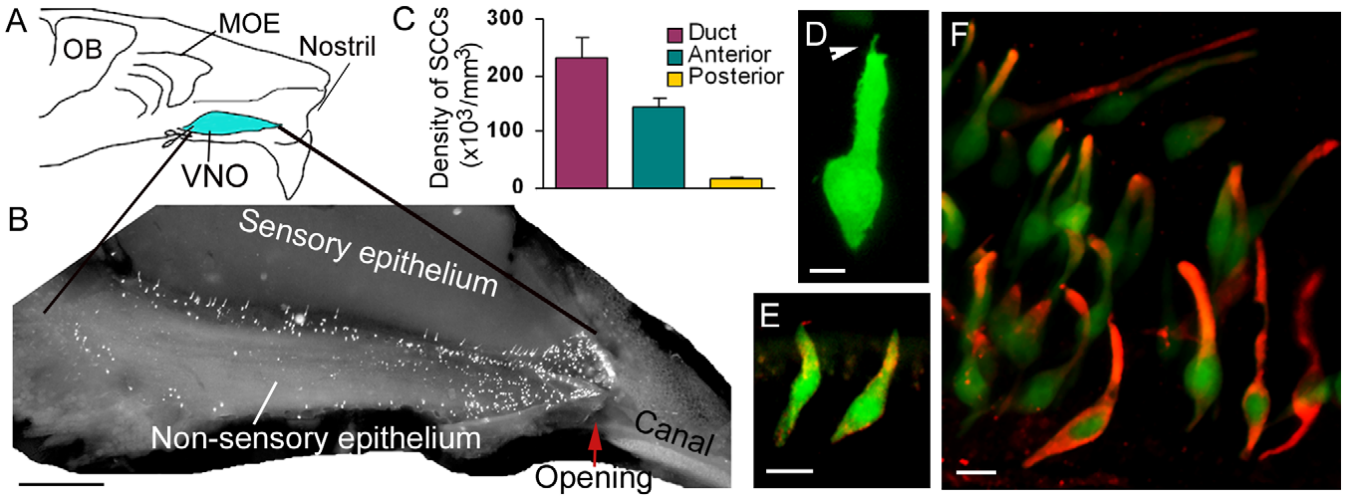
SCCs preferentially locate at the entrance duct of the VNO and express chemosensory signaling components. A: A schematic drawing of a mouse hemi-nose. MOE: main olfactory epithelium; OB: olfactory bulb. VNO: vomeronasal organ (blue). B: Luminal view of the entire non-sensory epithelium and entrance duct of a VNO from a TRPM5-GFP mouse. Bright spots are GFP-positive SCCs. Arrow points to the anterior opening. Anterior to the VNO, the cartilaginous stenonii canal channels external fluids to the VNO opening. C: Plot of SCC density at different regions as determined from horizontal VNO sections of four mice (Mean ± SEM), showing that the GFP-expressing SCCs preferentially reside at the entrance duct and adjacent 0.5 mm long anterior non-sensory epithelium. D: Confocal image of a typical GFP-expressing SCC. Arrowhead points to an apical microvillus. E: Immunolabeling of TRPM5 (red) in GFP-expressing cells (green) in a VNO section. F: Image taken from an epithelial strip from the entrance duct, showing that TRPM5 (GFP) expressing SCCs immunoreacted to an anti-α-gustducin antibody (red). Scales: B, 0.5 mm; D, 5 µm; E and F, 20 µm.

The VNO is situated at the ventral floor of the nasal cavity (Fig. 1A). Its narrow entrance duct is the only passage allowing chemical fluids to access the VNO. We exposed the luminal wall by cutting individual VNOs of TRPM5-GFP transgenic mice longitudinally to determine the distribution of the GFP-expressing SCCs (Fig. 1B). The entrance duct measured about 0.4 mm in length (n = 4). We found abundant GFP-positive cells at the entrance duct and the adjacent anterior non-sensory epithelium, with highest density being found at the entrance duct. Some SCCs were also found in the non-sensory epithelium of the posterior regions, but the density is approximately 13 times lower than that of the entrance duct as determined from VNO tissue sections of four mice (Fig. 1C). When counter-stained with the nucleus marker 4′,6-diamidino-2-phenylindole (DAPI), SCCs at the duct constitute approximately 20% of the total epithelial cells. Thus, these SCCs reside in a critical position to monitor fluid chemicals destined to the VNO lumen.

### SCCs of the VNO express key chemosensory-signaling proteins

The Morphology of the GFP-positive SCCs found in the VNOs were similar to the TRPM5-expressing SCCs in the respiratory epithelium [35], showing apical microvilli reaching the luminal surface and no axons emanating from the basal region (Fig. 1D). An anti-TRPM5 antibody positively immunolabeled these cells, confirming the expression of TRPM5 (Fig. 1E). Further, we immunolabeled VNO tissue sections and epithelial strips with an antibody against α-gustducin, a key element in taste sensation [31], [41]. We found that approximately 95% of TRPM5-expressing SCCs co-expressed α-gustducin. There was no apparent difference in the percent of cells showing co-localization in various regions of the VNO. We thus pooled the data (233 cells counted from various regions of non-consecutive tissue sections and strips from the VNOs of three mice; Fig. 1F). The expression of TRPM5 and α-gustducin strongly indicate chemosensibility of the SCCs.

### Trigeminal peptidergic nerve fibers appear to innervate TRPM5-expressing SCCs

**Figure 2:**
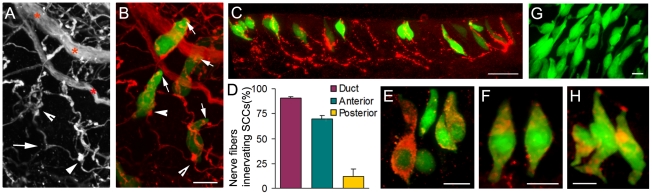
Trigeminal innervation and immuno-expression of ChaT and VAChT for ACh synthesis and packaging in SCCs. A: Confocal image of a VNO epithelial strip from a TRPM5-GFP mouse, showing both PGP 9.5-labeled trigeminal nerve bundles (asterisks) at basal lamina and many fine intraepithelial fibers. Arrowheads point to varicosities found typically in peptidergic fibers. Arrow points to a branching nerve fiber. B: The GFP-fluorescence image overlaid with (A). All TRPM5-expressing SCCs are apposed or wrapped closely by one or a few intraepithelial nerve fibers. Arrows point to apexes of SCCs. C: immunolabeling of substance P in a section of the anterior non-sensory epithelium. Note that most of the labeled intraepithelial fibers appear to innervate SCCs. D: Percentages of intraepithelial fibers innervating SCCs. E and F: Confocal images of TRPM5-expressing SCCs (green) immunoreacted to antibodies against the ChAT and VAChT respectively (red). G: Whole-mount fluorescence image of the ChAT (GFP)-expressing cells taken from a VNO entrance duct of a ChAT(BAC)-eGFP mouse. H: CHAT (GFP)-expressing cells of the VNO immunolabeled by the anti-α-gustducin antibody (red). Scales: B, F, G, and H, 10 µm; C, 50 µm; E, 20 µm.

Trigeminal fibers innervate the nasal mucosa and VNO non-sensory epithelium. The intraepithelial fibers are generally considered to be free nerve endings [30], [42]. We examined trigeminal innervation of the vomeronasal SCCs by labeling trigeminal fibers in epithelial strips and sections using an antibody against neuronal marker PGP 9.5. PGP 9.5-labeled fibers apposed SCCs closely, either coursing along or wrapping the SCCs (Fig. 2A and B). The immunolabeling of substance P, which labels the trigeminal peptidergic fibers, mimicked this result (Fig. 2C). We found 98.4% of the SCCs (pooled from various regions of the VNOs) were closely apposed by substance P positive fibers (876 cells examined from five mice). Interestingly, individual SCCs were apposed by one or a few intraepithelial nerve fibers and a single fiber sometimes contacted a few SCCs. We calculated the percent of intraepithelial trigeminal peptidergic nerve fibers innervating SCCs based on a simplified innervation pattern of one fiber per SCC. Unexpectedly, at the entrance duct nearly all the intraepithelial peptidergic fibers innervated the TRPM5-expressing SCCs (Fig. 2D). Thus it is most likely that at the entrance duct the SCCs detect chemicals and relay information onto trigeminal fibers.

### The SCCs are capable of synthesizing and packaging acetylcholine (ACh)

We determined whether the SCCs express ChAT and VAChT, two critical elements for synthesis and packaging of ACh, a potential transmitter. Similar to the TRPM5-expressing taste receptor cells [43], antibodies against ChAT and VAChT labeled the TRPM5-expressing SCCs in VNO tissue sections (Fig. 2E and F). Consistently, in transgenic mice, where the ChAT promoter drives the GFP expression [ChAT (BAC)-eGFP], we found that there were abundant GFP-positive cells in the VNOs (Fig. 2G). These cells resembled TRPM5-expressing SCCs in morphology, distribution and expression of α-gustducin (Fig. 2H). Thus, the TRPM5-expressing SCCs are capable of synthesizing and packaging ACh into vesicles and likely release ACh upon chemical stimulation.

### SCCs respond to a variety of chemical stimuli

**Figure 3:**
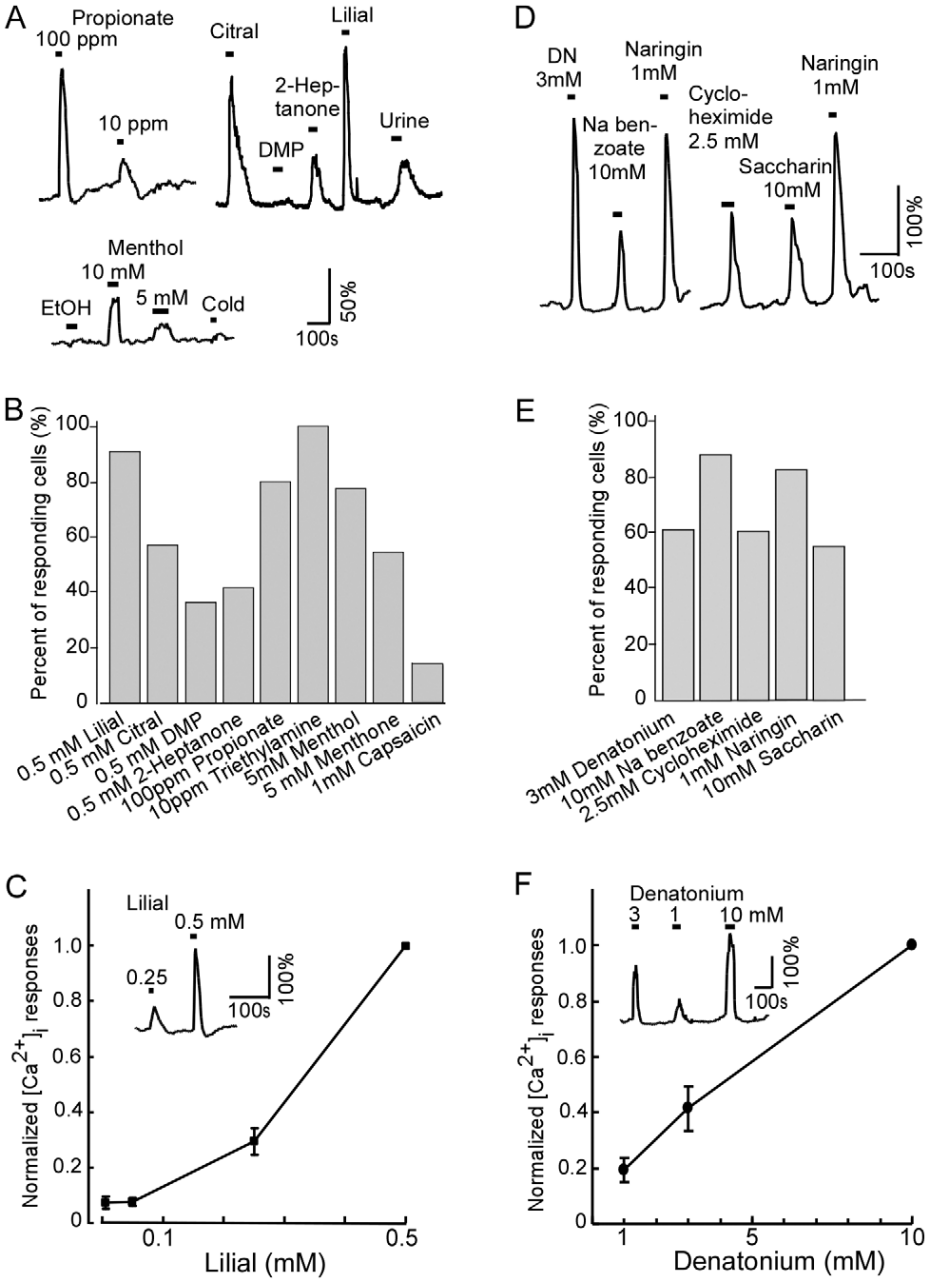
Chemical stimuli-induced changes in intracellular Ca^2**+**^ in isolated SCCs. A: Representative traces from three isolated SCCs responding to various stimuli with increases in intracellular Ca^2+^ levels. Odorous stimuli, 0.5 mM or otherwise indicated. Mouse urine (1∶100 dilution). Horizontal bars indicate stimulation periods. Note stimulus- and concentration-dependence of the response amplitudes. Ethanol (EtOH, 0.5%): solvent for menthol, cold: 4°C saline. B: Percentage of SCCs responding to odorous stimuli (n = 4 to 22). C: Concentration-dependent responses to lilial. Each SCC was challenged by lilial from 0.025 to 0.5 mM. The responses were normalized to the peak response value of 0.5 mM (n = 4, mean ± SEM). Inset, response traces of lilial at two different concentrations. D: Typical intracellular Ca^2+^ response traces to bitter-tasting compounds. DN: denatonium benzoate. E: Percentage of responding SCCs to bitter stimuli (n = 8 to 13). F: Concentration-dependent responses to denatonium (1, 3, 10 mM). The responses were normalized to the peak response value of 10 mM (n = 7, mean ± SEM). Inset, response traces of denatonium at three different concentrations. Vertical scales in A, C, D and F indicate percent changes from the resting Ca^2+^ levels.

In taste receptor cells, stimulation of bitter, sweet and umami substances elevates intracellular Ca^2+^ levels, leading to activation of TRPM5 [44]. We utilized the GFP expression in both TRPM5-GFP and ChAT(BAC)-eGFP mice to identify SCCs isolated from VNO tissues in Ca^2+^-imaging experiments. We reasoned that in natural conditions SCCs of the VNO would encounter both volatile and non-volatile substances in the fluids destined to VNOs. A wide variety of volatile chemicals can gain access to the VNO as either natural constituents of bodily secretions, environmental contaminants, or inhaled volatiles diffusing into nasal fluids. We therefore tested various individual odor chemicals including lilial and citral (plant product), propionic acid (a bacterial product found in animal skins), triethylamine (airborne irritant) [45], 2-heptanone and 2,5-demethylpyridine (DMP) (urinary pheromones), as well as mouse urine (a complex bodily secretion). In general, SCCs responded to odorants at high concentrations and the Ca^2+^ response amplitudes were concentration-dependent (Fig. 3A). SCCs that responded to at least one of the stimuli tested were used to calculate percent responding cells (Fig. 3B). Response profiles of individual cells are shown in table S1. We found that high percentage of SCCs responded to lilial (0.5 mM, 20 of 22 cells), propionate (10 – 100 ppm, pH adjusted to 7, 4 of 5 cells), and triethylamine (1 ppm, 4 of 4 cells). Interestingly, diluted mouse urine (1∶100), 2-heptanone and DMP at 0.5 mM induced smaller responses as compared to the lilial responses of the same cells. Fig. 3C shows lilial-induced responses that were concentration-dependent. These data demonstrate that odorous irritants are potent stimuli for SCCs of the VNO.

Also, we examined Ca^2+^ responses to capsaicin and menthol known to stimulate trigeminal free nerve endings [30], [46]. Only one out of seven SCCs tested responded to 10 -100 µM capsaicin (Fig. 3B). Interestingly, menthol at 1–5 mM induced responses in 31 of 34 cells (Fig. 3A and B). Menthone (1 mM), a chemical related to menthol, induced Ca^2+^ changes in 8 of 11 cells. Thus, lipophilic capsaicin is not a potent stimulus for SCCs.

We next examined responses of the SCCs to bitter-tasting substances. Taste cells detect bitter substances to avoid intake of toxins. We selected denatonium benzoate (a potent synthetic bitter compound commonly used in rodent taste studies), sodium benzoate (food preservative), cycloheximide (a natural product of bacterium *Streptomyces griseus*, also an inhibiter for protein biosynthesis*)*, naringin (a natural bitter compound in the grapefruit skin), and saccharin (artificial sweetener with bitterness at high concentrations). Bath application of each bitter substance (1 to 10 mM) induced rapid increases in intracellular Ca^2+^ levels (Fig. 3D). The percent of responding SCCs for individual compounds ranged from 56 to 88% (Fig. 3E; n = 6–62 for each substance; response profile in Table S2). Interestingly, individual SCCs responded to several, but not all the bitter compounds tested, indicating certain specificity. Responses to denatonium benzoate at concentrations of 1, 3, and 10 mM are plotted in Fig. 3F, showing concentration-dependence. Thus, SCCs of the VNO are capable of detecting a variety of bitter and toxic substances.

### Phospholipase C (PLC) signaling pathway is involved in the chemical responses in SCCs

**Figure 4:**
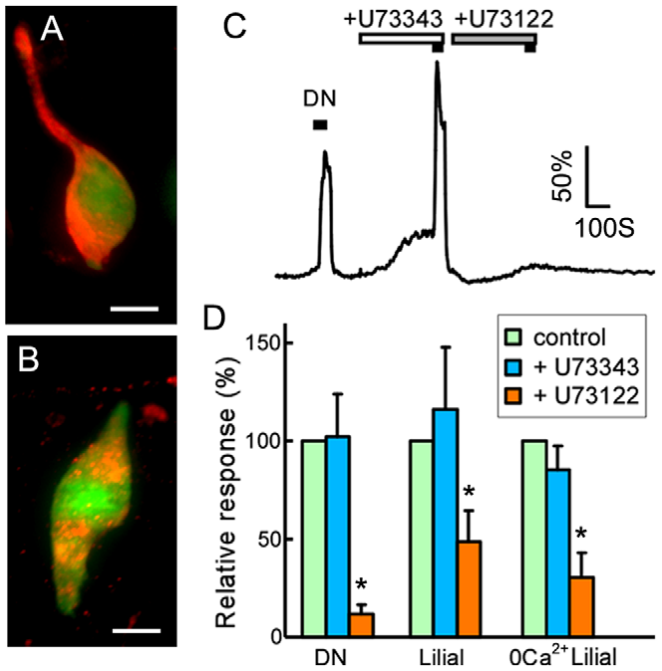
Involvement of the PLC signaling pathway. A and B: SCCs (green) of the VNO immunoreacted to antibodies against G-protein γ13 and PLCβ2 (red) respectively. Scales: 10 µm. C: The PLC inhibitor U73122 (5 µM), but not the negative control U73343 (5 µM), suppressed the denatonium (3 mM)-induced Ca^2+^ increase. D: Summary of the inhibition. The concentration of denatonium and lilial were 3 mM and 0.5 mM respectively. The peak responses of single SCC obtained in the presence of U73122 or U73343 were normalized to the control responses. The U73122 inhibition of responses to denatonium and lilial was statistically significant as compared to the controls (marked by the asterisks; n = 9 for denatonium, 7 for lilial, and 4 for 0 Ca^2+^ lilial).

In taste receptor cells, TRPM5 is activated by the PLC pathway [47]. We found SCCs of the VNO positively immunoreacted to antibodies against PLCβ2 and γ13, a G-protein γ subunit required for the PLCβ2 activation [48] (Fig. 4A and B). In Ca^2+^ imaging, the PLC inhibitor U73122 (5 µM), but not the negative control U73343 (5 µM), strongly suppressed the Ca^2+^ responses induced by denatonium and lilial (*t*-test, p = 0.0002 for denatonium, 0.0318 for lilial, n = 5−9; Fig. 4C). The suppression on the lilial responses was smaller than on the denatonium responses. To determine whether the Ca^2+^ increases were due to the Ca^2+^ release from internal Ca^2+^ stores via activation of the PLC signaling pathway, we replaced extracellular solution with nominally Ca^2+^ free saline (0 Ca^2+^), and tested again the U73122 and U73343 inhibition on lilial responses. The results were similar to those that were obtained in normal extracellular Ca^2+^ solution (Fig. 4D). These data suggest that the PLC pathway is involved in SCCs-mediated detection of bitter compounds and odorous irritants. The incomplete suppression, especially on the response to lilial, suggests that other mechanisms independent from the PLC pathway in SCCs likely are also involved.

### Chemical access to the VNO is regulated

**Figure 5:**
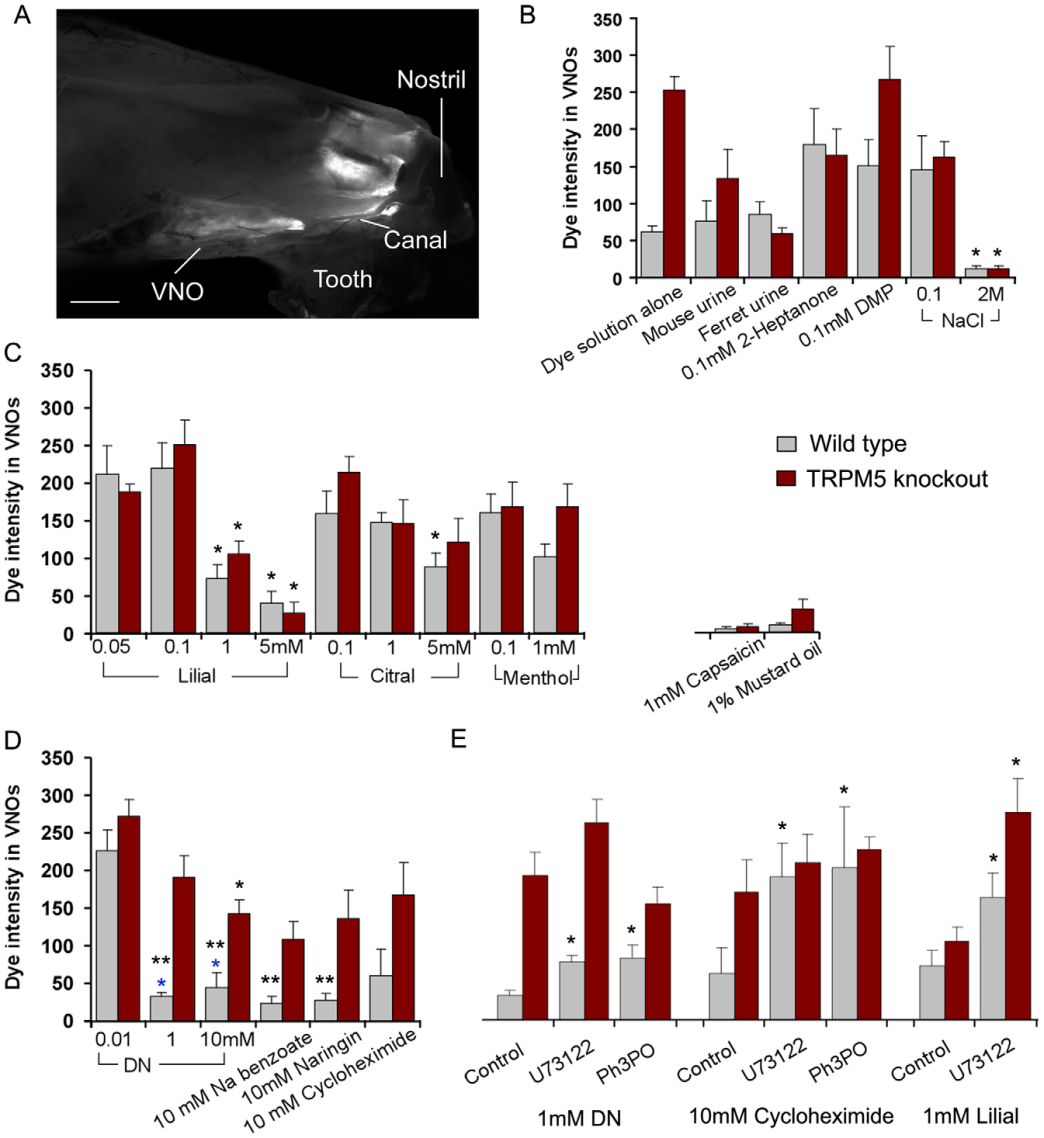
Chemical access to the VNOs of wild type and TRPM5 knockout mice. A: A representative fluorescence image of a hemi-nose taken after the dye assay, showing rhodamine fluorescence in the VNO and anterior nasal mucosa. Scale: 1mm. B, C, D, and E: Plots of averaged fluorescence intensity values in VNOs measured after the mixture applications (mean ± SEM). N = 5 to 14 animals for each group. Single asterisk: statistically significant as compared to the value of the lowest concentration of the same stimulus (B, C, and D) or to the control value (E). B: Applications of natural complex stimuli, synthetic pheromones and NaCl of 0.1 and 2M. C: Odorous stimuli applied at different concentrations, showing negative correlation between the intensity values and concentrations. Note very limited access of the dye-mixtures of capsaicin and mustard oil. These stimuli are known to directly stimulate trigeminal free nerve endings at the nostrils. D: Application of bitter-tasting stimuli. Denatonium (DN) was tested at three different concentrations, showing concentration-dependent mixture access. Note the significant differences between the WT and TRPM5 KO mice in the access of the bitter compounds at high concentrations (double asterisks). E: Changes in chemical access induced by local pharmacological inhibition of PLC and TRPM5. PLC inhibitor U73122 (10 µM) or TRPM5 inhibitor Ph_3_PO (100 µM) was applied to the VNOs using the delivery method in the dye assay. Inhibition of either PLC or TRPM5 significantly increased the access of bitter compounds to the VNOs in wild type animals. The access of dye-lilial mixture in both wild type and TRPM5 knockout mice was increased after U73122 treatment.

Our Ca^2+^ imaging study strongly indicated that the SCCs of the VNO detected both odorous irritants and bitter-tasting substances. However, it has never been documented whether the chemoreception of fluid constituents regulates chemical access to the VNO. To estimate the amount of stimulus fluid in the VNO lumen, we initially adapted a dye assay [19], in which we added rhodamine dye (8 µM) to the stimulus solutions, pipetted the mixtures onto the floors and walls of the animal cages, and allowed the mice to sample freely. We found that the VNOs fluoresced if the animals' noses made contact with the mixtures (data not shown). While our results are consistent with the previous publication [19], we found that the mice were not always interested in making contact with the samples, even those containing urine of the opposite gender. We then developed a method to apply mixtures directly and reliably to the snouts of behaving mice (5 µl in total for each animal). Using the new method, we found rhodamine fluorescence in the anterior nasal epithelium and VNOs (Fig. 5A). In no case did we find any rhodamine fluorescence in the posterior nasal epithelium including the main olfactory epithelium in all the dye assay experiments. The method was very reliable; nearly all the applications resulted in fluorescent VNOs. However, fluorescence intensity varied depending on the stimulus mixtures, indicating different amounts of dye-stimulus fluids entered the VNOs. This method thus allows us to evaluate carefully whether chemical stimuli and their concentrations influence the amount of chemical fluid accessed the VNO. In general the method resembles putting food in the mouth and allowing taste receptor cells to evaluate its contents in order to regulate food intake and toxin avoidance.

Initially, we examined the access of dye mixtures containing natural complex stimuli and synthetic pheromones to the VNO. Surprisingly, application of dye-urine mixtures, in which urine was either from mice or ferrets, only resulted in moderate fluorescence intensity in the VNOs as compared to the mixtures containing 100 µM pheromones 2-heptanone or DMP (Fig. 5B). The data indicate that other constituents that are nonspecific to vomeronasal neurons, such as high concentrations of volatiles and salts in urine samples might be detected to limit the sample access. We applied dye-NaCl mixtures. NaCl at 0.1M, an approximate concentration of Na^+^ in nasal surface liquid [49], generated much stronger fluorescence in VNOs than NaCl at 2M (n = 5 for each group), indicating that the high concentrations of salts, which likely are present in aged urinary deposits and other bodily secretions in natural conditions, can be detected to limit the fluid access. There was no significant difference between the wild type and TRPM5 knockout mice, suggesting functional expression of TRPM5 in SCCs is not critical for detecting Na^+^ salt or volatile compounds present in urinary samples. We also tested the dye solution alone. The VNO fluorescence intensity values measured from wild type animals were significantly lower than those from the knockout mice (n = 9−14). However, as shown in the above and the following experiments, the dye did not act as a dominant factor to influence the fluid access when mixed with other chemicals. These initial observations indicated complexity in the mechanisms controlling VNO chemical access, in which not only vomeronasal pump activation, but also sensory detection of chemical constituents is important.

We next tested the access of odorous chemicals at various concentrations because Ca^2+^ responses to these chemicals in SCCs were concentration-dependent. In all three odorous chemicals tested, lilial, citral, and menthol, the fluorescent intensity values decreased when concentrations of the stimuli were increased, showing negative correlation between the chemical concentrations and the amounts of the mixtures in the VNOs (Fig. 5C). To lilial, the intensity values at 1 and 5 mM were significantly lower than at 100 µM (ANOVA, F_5, 34_ = 16.273, P = 0.001 post hoc Tukey p<0.001). The reduction associated with higher concentrations of menthol was not significant, which may reflect a different potency of irritation. There is no significant difference between the wild type and knockout mice in this set of experiments (Fig. 5C). The results demonstrate that odorous fluid constituents are monitored largely by TRPM5-independent pathways to regulate the fluid access to VNOs.

We also tested capsaicin and mustard oil using this dye-assay. Unlike all other compounds tested, application of capsaicin (1 mM) and mustard oil (1%) did not result in measurable fluorescence in most of the VNOs. A few animals showed only very faint fluorescence in the VNOs after application of these stimuli (two out of six wild type and two out of five TRPM5 knockout mice for capsaicin; and two out of five for wild type and for knockout mice for mustard oil; Fig. 5D). These results together with the data from Ca^2+^ imaging strongly suggest that capsaicin and mustard oil are detected by free nerve endings before reaching the VNO entrance.

We next tested bitter-tasting compounds. In our Ca^2+^ imaging, denatonium benzoate, sodium benzoate, cycloheximide, naringin, and saccharin induced robust Ca^2+^ responses in SCCs of the VNO (Fig. 3). We first tested the access of denatonium-containing dye-mixtures and the concentration dependence of the access. At 0.01 mM, there seemed no limitation on the mixture access because the fluorescence intensity values measured in both wild type and TRPM5KO animals were high. However, at 1 mM and 10 mM, the intensity values decreased dramatically in wild type animals, suggesting significant reduction in the fluid access (ANOVA, F_5, 34_ = 27.76, P = 0.001, post hoc Tukey p<0.001). The reduction in the knockouts was moderate and significant only at 10 mM (post hoc Tukey p<0.001; Fig. 5D). Further the intensity values for 1 mM and 10 mM denatonium were significantly different between wild type and knockout animals (*t*-test, p = 0.005, 0.004 respectively), strongly suggesting that dysfunction of TRPM5 disrupts this regulation. We then tested other bitter-tasting compounds. Similar to the results of denatonium, there were significant differences in the intensity values between wild type and knockout animals for cycloheximide, sodium benzoate, and naringin at concentrations tested (Fig. 5E, *t*-test, p = 0.008, 0.002, 0.005 respectively). Since TRPM5 is only expressed in SCCs, not in the trigeminal fibers, our results strongly demonstrate the role of SCCs and TRPM5 in detecting bitter fluid constituents to limit their access to VNOs.

Because TRPM5 and PLC are also expressed in the main olfactory epithelium [37], [38], [50], we determined whether the regulation indeed is mediated by SCCs of the VNO. Using the same delivery method in the dye assay, we locally applied the TRPM5 blocker triphenyl phosphine oxide (Ph_3_PO; 100 µM) and PLC inhibitor U73122 (10 µM) to the VNOs. Pretreatment of Ph_3_PO or U73122 significantly increased the amount of the denatonium (1 mM)-dye mixture in the VNOs of wild type animals (133.71% and 149.91% respectively; n = 6−11, *t*-test, p = 0.002, 0.001 respectively; Fig. 5E). Similarly, pretreatment of these inhibitors increased dramatically the amount of cycloheximide (10 mM) entered the VNOs (231.13% and 211.42% respectively; n = 5, *t*-test, p = 0.048, 0.040 respectively). The changes in the TRPM5 KO mice were not statistically significant. In addition, we tested the effect of U73122 on odorous chemicals, since U73122 inhibited the Ca^2+^ responses to lilial in isolated SCCs. U73122 treatment significantly increased the amount of 1 mM lilial-dye mixture in VNOs of both wild type and knockout mice (n = 5 each group, *t*-test, p = 0.044). In control experiments, we mixed individual inhibitors with the dye, delivered them to the nostrils and monitored the fluorescence in the VNOs and nasal epithelium. Similar to other mixtures applied, the rhodamine fluorescence was found in the anterior nose and the VNOs (supporting Fig. S1A and B). We did not observe any fluorescent dye-inhibitor mixtures in the main olfactory epithelium in all the animals tested (n = 5 for each group). There was no significant difference in the fluorescence intensity between the wild type and TRPM5 KO mice (supporting Fig. S1C). These data strongly support the important role of the PLC pathway in SCCs in detecting bitter and odorous chemicals to regulate their access to VNOs. This result also suggests the presence of additional down-stream effecters of the PLC signaling pathway that are independent of TRPM5 in SCCs or other potential sensory apparatus.

## Discussion

Our results have uncovered that chemoreception of certain chemical constituents regulates the access of chemical fluids to the VNO. We have shown that there are abundant SCCs along the VNO entry passageway and they are capable of responding to a variety of odorous irritants and bitter-tasting compounds. Consistent with the results from the physiological recordings, our data obtained from the dye assay show that the amounts of the dye-stimulus mixtures allowed access to the VNOs are stimulus-dependent and inversely correlated to stimulus concentrations at ranges where SCCs responded. Further, we have shown that PLC is involved in both Ca^2+^ responses in isolated SCCs and in the regulation of stimulus access and that TRPM5 expressed in the SCCs is especially important for limiting the amount of bitter compounds entering the VNOs. Our results thus strongly suggest that the SCCs play an important role in detection of chemical constituents and regulation of chemical access.

**Figure 6:**
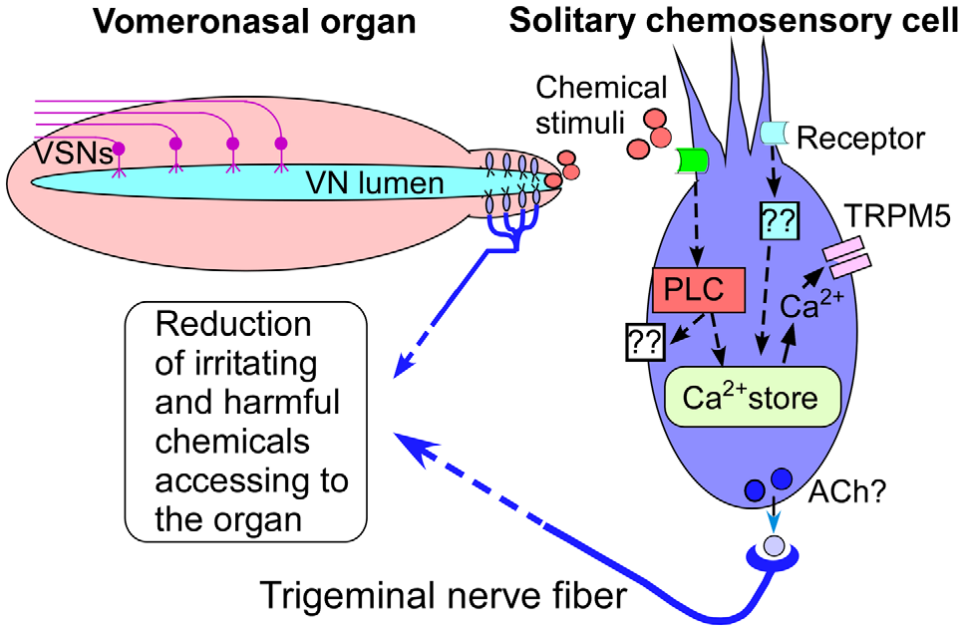
Schematic view of a novel sensory mechanism regulating chemical access to the VNO. The vomeronasal sensory neurons (VSN), which detect semiochemicals, are sequestered in the VNO, requiring chemical fluids to be drawn into the vomeronasal lumen via the anterior opening and entrance duct. SCCs reside densely at the entrance duct and detect odorous irritants and harmful substances in the stimulus fluids. Signal transduction in SCCs primarily involves the PLC signaling pathway, in which activation of PLC results in either an increase in intracellular Ca^2+^ levels via the internal Ca^2+^ stores, leading to activation of TRPM5 or activation of unknown effectors. PLC-independent pathway may also be involved in chemical responses. For sensory transduction of bitter compounds, the increase in intracellular Ca^2+^ opens TRPM5 ion channels. Activation of SCCs leads to release of ACh, a potential transmitter onto the trigeminal nerve fibers and consequently regulating the access of chemical fluids.

A schematic model of this chemoreception-mediated regulation is shown in Fig. 6. Similar to the pupillary light reflex [51], this regulation is initiated by a sensory mechanism independent from the primary sensory neurons, in this case, the vomeronasal neurons and controls the amount of stimuli entering the sensory organ. Most likely the entrance duct serves as a critical location for sensory detection of chemical constituents to take place because of the unique anatomy of the VNO. The VNO, enclosed by a thin layer of bony tissue, is isolated anatomically. This physical isolation, while it may serve to protect the vomeronasal neurons, requires chemical fluids to be drawn into the lumen. The rigidity of bony tissue enables vasomotor movement of the vomeronasal veins to change the luminal pressure, which acts as a pumping mechanism to draw in or expel chemical fluids, such as bodily secretions containing semiochemicals [22], [23], [24], [25]. These fluids are complex chemical blends and when deposited in the environment are often aged, degraded and contaminated before being drawn into the VNO. Chemoreception-mediated regulation of fluid access to the VNO, especially limiting harmful or contaminating substances, likely plays a very important role in protecting vomeronasal sensory neurons.

### Distribution and trigeminal innervation of SCCs in the VNO

SCCs are known to be present in the VNO from scattered information obtained from previous immunolabeling studies, which show that a subset of cells in the VNO express microvillar cell marker espin [52] as well as signaling proteins α-gustducin [31], TRPM5 [53], and type III IP3 receptor [52]. These results indicate that VNO SCCs express similar cellular components that are similar to other SCCs found in the nasal respiratory epithelium and other epithelial tissues [31], [32], [35], [54]. However, until now SCCs of the VNO have not been studied in detail, particularly in direct relation to the unique structure and function of the VNO.

The VNO actively pumps in complex chemical fluids for detection of semiochemicals. If the SCCs function to monitor certain chemical constituents in fluids before they reach the VNO lumen, it would be logical for these SCCs to reside along the passageway to detect a variety of chemicals and transmit sensory information to the nervous system, so that access of certain chemicals to the VNO lumen is regulated. Indeed, we found the highest density of SCCs at the entrance duct of the VNO, implying that SCCs may play a primary role in detecting chemical constituents before being drawn into the VNO lumen.

While trigeminal free nerve endings are known to be present in the entrance duct and non-sensory epithelium of the VNO [30], [42], there is no published study showing these trigeminal nerve fibers serve to directly detect chemical irritants or signal tissue damage and inflammation. We immunolabeled trigeminal peptidergic fibers and estimated the percent of trigeminal fibers innervating the SCCs in the VNO. Our data clearly showed that a great majority of the trigeminal intraepithelial fibers appear to innervate the SCCs at the entrance duct. This result was unexpected, strongly suggesting that at the entrance duct the trigeminal fibers receive sensory information from the SCCs, although further experiments are needed to determine signal transmission and whether the SCCs release ACh or other molecules as neurotransmitters upon stimulation.

Our results also reveal that the intraepithelial nerve fibers and the innervated SCCs are not in one-to-one relations. Individual SCCs sometimes were apposed by more than one fiber and an intraepithelial trigeminal fiber could branch to appose two or a few SCCs. How this pattern of innervation would influence the coding of the sensory information is not known. In this study we could only estimate the percent of nerve fibers innervating the SCCs based on one fiber per SCC. Precise determination, which would require confocal imaging and reconstruction of the entire region, is beyond the scope of this study. Also further examination will be needed to confirm the involvement of trigeminal system.

### Chemical stimuli-evoked Ca^2+^ responses in SCCs

In our Ca^2+^ imaging study, SCCs isolated from VNOs responded to a variety of chemicals at certain concentrations, including urine samples, pheromone, odorants, bitter-tasting substances and trigeminal irritants. The Ca^2+^ imaging results suggest that the SCCs of the VNO are broadly tuned, although certain specificity could be seen as individual SCCs generally responded to several but not all the stimuli applied. The responses were concentration-dependent and the percent of responding cells varied significantly among chemicals, indicating that the SCCs are highly responsive to some chemicals such as triethylamine but less sensitive to the others. Interestingly, capsaicin, a well known lipophilic trigeminal stimulus, rarely induced responses in SCCs. Currently whether the SCC responses to odorous chemicals are receptor mediated remains to be determined. More than half of SCCs responded to various bitter compounds that differ in structure. This is consistent with published results that SCCs in the respiratory epithelium express bitter receptors and respond to bitter compounds [32], [34], [36]. The broad responsiveness of the SCCs at the entrance duct may be advantageous for monitoring a variety of chemicals in the fluids destined to the VNO, because of the complexity of the natural stimuli and the potential for contamination by a wide range of chemicals when bodily secretions are deposited in the environment.

Our Ca^2+^ imaging study also provides insight into the sensory transduction pathway of the SCCs. Consistent with our immunolabeling results showing the presence of PLC and γ13 in SCCs, we found that application of PLC inhibitor suppressed both responses to bitter and odorant compounds significantly in our Ca^2+^ imaging study. Interestingly, the percent inhibition for bitter compounds was higher than that for the odorous lilial. Because of the incomplete inhibition, it is likely that PLC-independent pathways also are involved. Further studies are needed to determine these mechanisms.

### Regulation of chemical access to the VNO

Our fluorescence dye assay allowed us to gain insight into whether chemical access to the VNO is regulated. We found surprisingly that only moderate amounts of dye-urine mixtures were drawn into the VNOs as compared to the other mixtures containing a single stimulus at low concentration, indicating that the access of complex stimuli is regulated. Consistently, we found that isolated SCCs responded to diluted urine samples in the Ca^2+^ imaging experiment. Whether this regulation would prevent vomeronasal sensory neurons from being stimulated excessively is not known, since the sensory neurons are very sensitive and can detect semiochemicals in nano- to micro-molar ranges [14], [55], [56]. The access of high concentrations of salts and volatiles to the VNO was found to be limited. This regulation likely plays a role in limiting non-specific stimuli that are commonly present in complex bodily secretions, so that the proper luminal environment can be maintained. In addition, bodily secretions deposited in the environment usually undergo aging and degradation and often are contaminated. This regulation also would prevent irritants and contaminants from entering the VNO and causing damage. Indeed, we found that the access of dye-stimulus mixtures to the VNOs was reduced when the mixtures contained either odorous irritants or bitter compounds. Higher concentration of irritants or bitter compounds generally resulted in greater reduction of access. This is consistent with the notion that high levels of odorants are irritants to animals and humans [28], [29] and that bitter compounds generally are treated as toxic.

Results from the dye assay also revealed that there is a significant difference in the role of TRPM5 in regulating the access of odorous versus bitter compounds. For the odorous chemicals tested, there was generally no difference in the amounts of stimuli entered the VNOs between TRPM5 knockout and wild type animals. However, for the bitter compounds, except at the lowest concentration tested, the access to the VNO in the knockout mice was significantly higher than the wild type mice, suggesting the regulation is deficient in knockout animals. Application of TRPM5 inhibitor produced similar effect. Together, these results demonstrate the important role of TRPM5 in chemoreception-mediated limitation of the access of bitter compounds. It is likely that TRPM5 is not the sole signaling ion channel mediating the bitter signal transduction in the SCCs of the VNO, since access of denatonium at 10 mM concentration was also reduced in the knockout animals.

Similar to the TRPM5 inhibitor, application of the PLC inhibitor U73122 also disrupted the regulation on access to the VNO of bitter compounds in wild type but not in TRPM5 knockout mice. Unlike the TRPM5 inhibitor, U73122 also disrupted the regulation on the access of lilial in both wild type and TRPM5 knockout mice. These data are consistent with the results obtained from our Ca^2+^ imaging, indicating the important role of the PLC pathway and presence of additional TRPM5-independent down-stream effecters.

### Role of SCCs in regulating chemical access to the VNO

Several lines of evidence strongly suggest that SCCs play an important role in regulating chemical access. First and the most striking evidence was obtained from TRPM5 knockout mice and pharmacological inhibition in the dye assay, which clearly show the importance of TRPM5-expressing SCCs in detecting bitter substances to limit their access to the VNO. TRPM5 is not expressed in the trigeminal nerve fibers innervating the SCCs. Second, the chemical response profiles of SCCs correlate with the regulation on the chemical access. Chemical stimuli at concentration ranges that induced intracellular Ca^2+^ responses in SCCs also triggered the regulation and had their access limited. Third, the PLC inhibitor U73122 suppresses the stimulus-induced Ca^2+^ responses in SCCs as well as disrupts the regulation on the access of such chemicals. Finally, the appropriate location of SCCs at the entrance duct also supports the role of these SCCs. Because chemicals that SCCs responded to are potentially irritating and toxic, it is plausible to consider that the sensory information provided by the SCCs is used primarily to limit the VNO access of such chemicals. However, the sensory information may also facilitate the expulsion of such chemical fluids once they have entered the VNO. Clearly, SCCs do not detect all the irritating and harmful chemicals and therefore other sensory mechanisms are likely involved, such as the trigeminal free nerve fibers. In our study, capsaicin, a highly lipophylic noxious chemical, hardly reached the VNOs, and SCCs rarely responded to it, indicating that capsaicin directly activates free nerve endings at the nostrils before reaching the VNOs.

In summary, our results strongly indicate the capability of SCCs in detecting potentially irritating and toxic chemical constituents to limit their access to the VNO. This supports the emerging role of SCCs in protecting vital organs. Because of the necessity of chemical intake, some chemical fluids likely would gain access to the VNO, despite containing irritating or bitter chemicals. Chemical access to the VNO thus reflects both the vomeronasal pumping activity and chemoreception-mediated regulation.

## Materials and Methods

### Animals

Adult wild type and genetically modified mice of C57BL/6 background were used. The original breeding pairs of TRPM5 GFP transgenic mice [57] and TRPM5 knockout (KO) mice [58] were provided kindly by Dr. RF Margolskee. The original breeding pairs of ChAT(BAC)-eGFP mice, where the eGFP was placed under the control of endogenous choline acetyltransferase (ChAT) transcriptional regulatory elements, were provided kindly by Dr. MI Kotlikoff [59]. Offspring were genotyped using the polymerase chain reaction. Adult mice of the same gender were housed in polycarbonate cages suspended on a rack with automatic water supply (2–5 mice per cage) in a facility with temperature, humidity and ventilation regulated. Both food and water were provided ad libitum. All animal care and procedures were approved by the Animal Care and Use Committees (IACUC) of University of Maryland, Baltimore County.

### Immunocytochemistry

#### Tissue preparation

Mice were anesthetized with Avertin (0.02 ml/g body weight), which was made up with 2.5 g 2,2,2 tribromoethanol, 5 ml 2-methyl-2-butanol in 200 ml 0.1 M phosphate buffer. Anesthetized mice were perfused transcardially with buffered fixative containing paraformaldehyde, L-lysine, and sodium m-periodate [60]. The nose was harvested and post-fixed for 1–2 hours. For direct visualization of GFP expression and location of SCCs, the nose was split along the midline and individual VNOs were opened longitudinally along the ventral conjunction of the sensory and non-sensory epithelia to expose the luminal surface of the VNO and entrance duct. For immunolabeling on tissue sections, bones surrounding the noses were removed and tissues were transferred into 0.1 M phosphate buffer saline (PBS) with 25% w/v sucrose overnight before being embedded with OCT (Sakura Finetek, Torrance, CA). Transverse or horizontal VNO sections (14 µm) were cut using a cryostat (Microm International, Walldorf, Germany), mounted onto Superfrost plus slides (Fisher Science, Pittsburgh, PA) and stored at −80°C degree until used.

#### Immunolabeling

VNO sections or epithelial strips were rinsed and incubated in blocking solution containing 2% normal donkey serum, 0.3% Triton X-100 and 1% bovine serum albumin in PBS for 1.5 hour. Sections were then incubated 12 to 72 hours with primary antibodies against each of the following proteins: TRPM5 (1∶250), γ13 (1∶500), both were provided kindly by Dr. RF Margolskee [61], α-gustducin (1∶1000, Santa Cruz Biotechnology, Santa Cruz, CA), substance P (1∶1000, Chemicon, Temecula, CA), PGP 9.5 (ubiquitin carboxyl-terminal hydrolase; 1∶500, Chemicon), ChAT (1∶100; Chemicon), PLCβ2 (1∶200; Santa Cruz Biotechnology), and VAChT (1∶200, Sigma). Sections were washed and reacted with secondary antibody conjugated either with Alexa 555, or Alexa 647, (Invitrogen, Eugene, OR) for 1 hour at room temperature before being washed and mounted on slides with Fluoromount-G (Southern Biotech, Birmingham, AL). Removing primary antibodies in control experiments resulted in negative labeling. The specificity of the TRPM5, ChAT and VAChT antibodies has been determined previously [35], [43]. Images were taken using Olympus compound epi-fluorescence microscopes BX 41 or BX 61 equipped with a spinning disk confocal unit (Olympus America, Center Valley, PA). In the cases involving dual fluorescent labeling, the serial acquisition mode was used for Z-step confocal images.

#### Determining the density of SCCs in the VNO

We examined the SCC density in the entrance duct, and the anterior and posterior non-sensory epithelium. The entrance duct was the most anterior region measured from the anterior opening to the beginning of the sensory neuro-epithelium. We divided the non-sensory epithelium lining the convex luminal wall into two regions, the anterior (0.5mm in length, measured from the end of the entrance duct) and posterior (the rest of epithelium). To estimate the SCC density at the entrance duct and adjacent anterior non-sensory epithelium, multiple Z-step confocal images with a 40x lens were taken to cover each region from horizontal VNO sections. The sections sampled were 14 µm thick, non-consecutive, and approximately 70 µm apart. The number of GFP cells was counted and the epithelial volume was calculated using the confocal Z-step distance multiplied by the area measured using the NIH Image J software (http://rsbweb.nih.gov/ij/). The cell density measured from images of the same regions of four animals was averaged. The SCC density in posterior non-sensory epithelium of the VNOs was determined from low-magnification epi-fluorescence images because of its low density.

#### Determining trigeminal innervation of SCCs

VNO sections from TRPM5 GFP mice were immunolabeled with substance P and confocal-imaged. We examined individual optical sections to determine the number of SCCs that were apposed closely by substance P-labeled intraepithelial nerve fibers. Intraepithelial nerve fibers that extended 3/4 of the epithelium thickness and did not appose any SCCs were counted as free nerve endings. The percentage of nerve fibers that innervate SCCs was calculated based on a simplified innervation pattern of one fiber per SCC using the following formula: percentage (%) = Number of innervated SCCs/(Number of free nerve endings + Number of Innervated SCCs)*100.

### Solutions and chemicals for Ca^2+^ imaging and fluorescent dye assay

Odorous chemicals were obtained form Aldrich Chemical Company Inc (Milwaukee, WI), Fluka (Ronkon Koma, NY), TCI America (Portland, OR) at the highest purity available. Odorants were made by dilution with vigorous vortexing and stocks of odorants were stored at −20°C. Menthol, menthone, capsaicin, and naringin were dissolved in EtOH as stock solutions. For Ca^2+^ imaging, the stock solutions were diluted with Tyrode's saline, which contained (in mM): 140 NaCl, 5 KCl, 10 *N*-2-hydroxyethylpiperazine-*N*'-2-ethanesulfonic acid buffer (HEPES), 1 MgCl_2_, 3 CaCl_2_, 10 Na pyruvate and 10 D-glucose (pH 7.4). EtOH itself in Tyrode's saline at the final concentration did not induce responses in Ca^2+^ imaging experiments. For the dye assay, odorants were diluted in distilled water containing 8 µM rhodamine 6G dye (Invitrogen). TRPM5 ion channel inhibitor triphenyl phosphine oxide (Ph_3_PO, Sigma) was prepared to a final concentration of 100 µM. The PLC inhibitor U73122 and its inactive analogue U73343 (Calbiochem, San Diego, CA) were dissolved in DMSO and diluted into the bath solution at a final concentration of 5 µM for Ca^2+^ imaging experiment and 10 µM for the dye assay. Final concentration of DMSO was <0.2%, which did not induce responses in Ca^2+^ imaging experiments.

### Ca^2+^ imaging

Fura-2 ratio imaging was used to measure intracellular Ca^2+^ levels [62], [63]. The method of isolating SCCs from VNOs was adapted from our previous study [37]. Briefly, the entrance and anterior VNOs was removed from TRPM5-GFP or ChAT(BAC)-eGFP mice after euthanasia, cut into small pieces and placed in Ca^2+^-Mg^2+^-free Tyrode's saline with 10–30 U/ml of papain (Worthington, Lakewood, N.J) and 2 mM cysteine for 30 min at room temperature. VNO cell dissociation was facilitated by gentle pipetting. The supernatant was transferred to an O-ring chamber on a cover slip pre-coated with concanavalin A (Sigma). After the cells were settled, the solution was replaced with Tyrode's saline containing 2 µM fura-2/AM (Invitrogen) for 20 minutes and washed with normal Tyrode's. Excitation wavelength was alternated between 340 and 380 nm using a filter wheel incorporated to a xenon lamp system (Lambda LS; Sutter Instruments, Novato, CA). The excitation light was guided via a liquid optical fiber to an inverted microscope (Olympus IX71). The ratio of fluorescence intensity at excitation wavelengths of 340 and 380 nm indicated the intracellular Ca^2+^ levels. Fluorescent images were obtained with a 40X oil UV objective lens (N.A. 1.3) with a 510±42 nm emission filter (Semrock: Rochester NY). Imaging Workbench software version 5.2 (INDEC BioSystems, Santa Clara, CA) was used to capture images and to change the position of the filters. Pairs of images at the two wavelengths were acquired every three seconds. In experiments where we determined the effects of the extracellular Ca^2+^ on stimulus-induced Ca^2+^ responses, nominal extracellular CaCl_2_ were achieved by omitting the CaCl_2_ from the Tyrode's saline. We considered a change in the intracellular Ca^2+^ levels (Ratio of F340/F380) to be a stimulus-induced response if the peak value of the change during stimulation was greater than two standard deviations above the mean resting level, which was obtained by averaging 10 data points (3 seconds each) before applying the stimulus in each cell tested [62], [63].

### Fluorescent dye assay

#### Stimulus delivery

Individual mice were gently transferred to a 12.7×7.6×5.1 cm (width × depth × height) closed box with a 1.27 cm square hole on a side wall. The mouse in the box was allowed to move around and explore freely. Because of the limited space, the mouse quickly turned its attention to the hole and began to chew on edges of the hole or protruded its nose through the hole, making the nostrils accessible. During this time, a droplet of the dye-stimulus mixture was applied to the nostrils and was drawn into the nose quickly by the mouse. This procedure was repeated several times until a total of 5 µl was applied. For each mouse the entire procedure of stimulus delivery usually lasted 3–5 min.

#### Inhibitor delivery

TRPM5 inhibitors Ph_3_PO (100 µM) and PLC inhibitor U73122 (10 µM) respectively were delivered to the noses of individual animals using the same method described in stimulus delivery. For each animal, a total of 5 µl inhibitor solution was applied. The mouse remained in the box for 15 minutes followed by application of the dye-stimulus mixture. For controls, we mixed the inhibitors with the rhodamine dye and delivered the mixture to the animals to monitor access of the inhibitors to the VNO and nasal cavity. All animals in this control experiment showed dye fluorescence in their VNOs. The dye-inhibitor solutions did not spread to posterior nasal mucosa.

#### Fluorescence imaging of dye-stimulus solution in VNOs

After the dye-stimulus mixture was delivered, the mouse was euthanized by CO_2_ followed by cervical dislocation. The blood was drained from the heart before the nose was removed and split to expose the VNOs. The nasal epithelium covering the VNOs was then removed, leaving the VNO intact and encapsulated by the thin layer of bones. For measurement of fluorescence intensity, images were taken using a 2x lens from ventral VNOs. At this magnification, the thin bone did not interfere with fluorescence imaging. Background images were taken from the respiratory epithelium where there was no dye staining. For control, some VNOs were opened longitudinally using a pair of fine scissors along the ventral conjunction of sensory and non-sensory epithelia. The luminal surfaces of the VNOs were imaged. We found one-to-one correlation with the results obtained in intact VNOs. The fluorescence intensity value for each VNO was measured using the NIH Image J software from which the background intensity value was subtracted. The values of the two VNOs of each mouse were combined and the values measured from five or more mice in each group were averaged.

### Statistical analyses

For comparison of Ca^2+^ imaging data, one-tailed Student's *t*-test was performed. For data obtained by the dye assay, one-way ANOVA with Tukey's post hoc test was used to compare the fluorescence intensity values across groups. One-tailed Student's *t*-test was also used to determine the significance of pharmacological inhibition and the differences between wild type and knockout mice of the same treatment. P<0.05 was considered to be statistically different.

## Supporting Information

Table S1 (0.06 MB DOC)

Table S2 (0.05 MB DOC)

Figure S1 Access of TRPM5 and PLC inhibitors to the VNOs of wild type and TRPM5 knockout mice. A and B: Representative fluorescence images taken from the hemi-noses of wild type and knockout mice respectively after application of the rhodamine dye-inhibitor mixtures. A: PLC inhibitor U73122 (10 µM). B: TRPM5 inhibitor Ph3PO. Note strong rhodamine fluorescence in the VNOs, especially at the entrance duct. There was no fluorescence in the main olfactory epithelium. Scale: 1mm. C: Plot of averaged fluorescence intensity values in VNOs measured after application of the dye-inhibitor mixtures (mean ± SEM). N = 5 animals for each group. There is no significant difference in the intensity values between wild type and KO mice.
(2.65 MB TIF)
