# Supplementary material for: Chemoreception Regulates Chemical Access to Mouse Vomeronasal Organ: Role of Solitary Chemosensory Cells
Source: PLoS One. 2010 Jul 30;5(7):e11924. doi: 10.1371/journal.pone.0011924 (PMC2912856; doi:10.1371/journal.pone.0011924)
Supplement: Table S1 — (0.06 MB DOC) [file pone.0011924.s001.doc]

**Table S1. Response profile for high concentration odorants at 0.5 mM**

| **Cell#** | **Lilial** | **Citral** | **DMP** | **2-Heptanone** |
| --- | --- | --- | --- | --- |
| 1 |  | **-** | **+** | **+** |
| 2 | **+** | **+** | **+** | **-** |
| 3 | **+** | **-** | **-** | **-** |
| 4 | **+** | **+** | **-** | **-** |
| 5 | **+** | **+** | **-** | **-** |
| 6 | **+** | **+** | **-** | **-** |
| 7 | **+** | **-** | **-** | **+** |
| 8 | **+** | **+** | **+** | **+** |
| 9 | **+** | **-** | **-** | **+** |
| 10 | **+** | **-** |  | **-** |
| 11 | **-** | **-** | **-** | **-** |
| 12 | **+** | **-** | **-** | **-** |
| 13 | **+** | **+** | **+** | **+** |
| 14 | **+** | **+** |  |  |
| 15 | **+** | **+** |  |  |
| 16 | **+** | **-** |  |  |
| Number of responded cells/total cells tested | 14/15 | 8/16 | 4/12 | 5/13 |
| % of cells responded | 93% | 50% | 33% | 38% |

Cells tested with two or more odorants are listed. +: responded.

-: no response. Blank: not tested.
