# Supplementary material for: Chemoreception Regulates Chemical Access to Mouse Vomeronasal Organ: Role of Solitary Chemosensory Cells
Source: PLoS One. 2010 Jul 30;5(7):e11924. doi: 10.1371/journal.pone.0011924 (PMC2912856; doi:10.1371/journal.pone.0011924)
Supplement: Table S2 — (0.05 MB DOC) [file pone.0011924.s002.doc]

**Table S2. Response profile for bitter compounds**

| **Cell#** | **Denatonium**  **benzoate** | **Na benzoate** | **Cycloheximide** | **Naringin** | **Saccharin** |
| --- | --- | --- | --- | --- | --- |
|  | 3 mM | 10 mM | 2.5 mM | 1 mM | 10 mM |
| 1 | **+** | **+** | **-** | **+** | **+** |
| 2 | **-** | **-** | **-** | **+** | **-** |
| 3 | **+** | **+** | **-** | **+** | **-** |
| 4 | **+** | **+** | **+** | **-** | **+** |
| 5 | **-** |  |  | **+** | **-** |
| 6 | **+** | **+** | **+** | **-** |  |
| 7 | **+** | **+** | **+** | **+** | **+** |
| 8 | **+** | **+** | **+** | **+** | **+** |
| 9 | **-** |  | **-** | **+** | **-** |
| 10 | **+** | **+** | **+** | **+** | **+** |
| Number of responded cells/total cells tested | 7/10 | 7/8 | 5/9 | 8/10 | 5/9 |
| % of cells responded | 70% | 88% | 56% | 80% | 56% |

Cells tested with 3 or more bitter compounds are listed in the table. +: responded.

-: no response. Blank: not tested.
