# Supplementary figures and images for: Chemoreception Regulates Chemical Access to Mouse Vomeronasal Organ: Role of Solitary Chemosensory Cells
Source: PLoS One. 2010 Jul 30;5(7):e11924. doi: 10.1371/journal.pone.0011924 (PMC2912856; doi:10.1371/journal.pone.0011924)

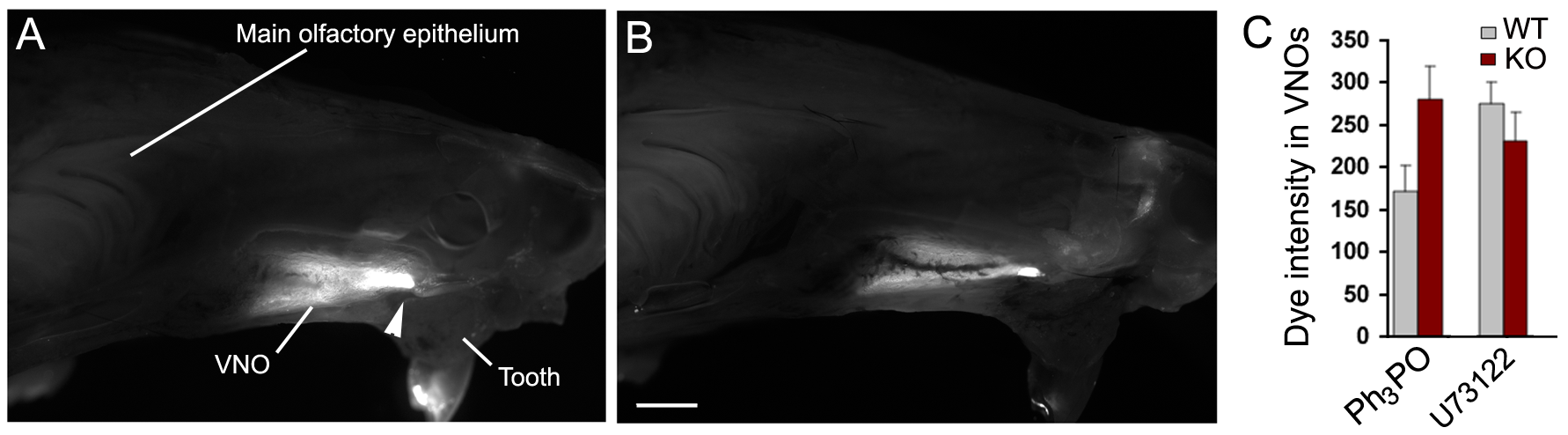

Supplement: Figure S1 — Access of TRPM5 and PLC inhibitors to the VNOs of wild type and TRPM5 knockout mice. A and B: Representative fluorescence images taken from the hemi-noses of wild type and knockout mice respectively after application of the rhodamine dye-inhibitor mixtures. A: PLC inhibitor U73122 (10 µM). B: TRPM5 inhibitor Ph3PO. Note strong rhodamine fluorescence in the VNOs, especially at the entrance duct. There was no fluorescence in the main olfactory epithelium. Scale: 1mm. C: Plot of averaged fluorescence intensity values in VNOs measured after application of the dye-inhibitor mixtures (mean ± SEM). N = 5 animals for each group. There is no significant difference in the intensity values between wild type and KO mice. (2.65 MB TIF) [file pone.0011924.s003.tif]
